# Dental and Craniofacial Anomalies Associated with Axenfeld-Rieger Syndrome with PITX2 Mutation

**DOI:** 10.1155/2010/621984

**Published:** 2010-03-21

**Authors:** Simone Dressler, Philipp Meyer-Marcotty, Nicole Weisschuh, Anahita Jablonski-Momeni, Klaus Pieper, Gwendolyn Gramer, Eugen Gramer

**Affiliations:** ^1^Department of Pediatric Dentistry, Dental School, University of Marburg, Georg-Voigt-Str. 3, 35033 Marburg, Germany; ^2^Department of Orthodontics, University of Wuerzburg, 97070 Wuerzburg, Germany; ^3^Molecular Genetics Laboratory, Universitiy Clinics Tuebingen, 72076 Tuebingen, Germany; ^4^University Hospital for Paediatric and Adolescent Medicine, 69120 Heidelberg, Germany; ^5^Department of Ophthalmology, University of Wuerzburg, 97080 Wuerzburg, Germany

## Abstract

Axenfeld-Rieger syndrome (ARS) (OMIM Nr.: 180500) is a rare autosomal dominant disorder (1  :  200000) with genetic and morphologic variability. Glaucoma is associated in 50% of the patients. Craniofacial and dental anomalies are frequently reported with ARS. The present study was designed as a multidisciplinary analysis of orthodontic, ophthalmologic, and genotypical features. A three-generation pedigree was ascertained through a family with ARS. Clinically, radiographic and genetic analyses were performed. Despite an identical genotype in all patients, the phenotype varies in expressivity of craniofacial and dental morphology. Screening for PITX2 and FOXC1 mutations by direct DNA-sequencing revealed a P64L missense mutation in PITX2 in all family members, supporting earlier reports that PITX2 is an essential factor in morphogenesis of teeth and craniofacial skeleton. Despite the fact that the family members had identical mutations, morphologic differences were evident. The concomitant occurrence of rare dental and craniofacial anomalies may be early diagnostic indications of ARS. Early detection of ARS and elevated intraocular pressure (IOP) helps to prevent visual field loss.

## 1. Introduction

Axenfeld-Rieger syndrome (ARS) (OMIM Nr.: 180500) is a rare autosomal dominant disorder (1  :  200000) with variable morphology characterized by malformations of the anterior segment of the eye such as Iris hypoplasia, iridocorneal adhesions, corectopia, polycoria, and embryotoxon posterius. Glaucoma is associated in 50% of the cases [1]. Craniofacial, dental, and umbilical anomalies are also regularly reported in connection with ARS [2, 3]. Characteristic craniofacial features are maxillary hypoplasia, hypertelorism, and telecanthus. Dental features include hypodontia/oligodontia of primary and permanent dentition, microdontia, short roots, taurodontism, and abnormally shaped teeth.

Other systemic features like anomalies of the pituitary gland, middle ear deafness, heart defects, hypospadia, short stature, and mental retardation were diagnosed in several ARS patients [1, 4, 5]. 

Three genetic loci have been associated with ARS so far. The genes *FOXC1* and *PITX2* encode transcription factors and are located on chromosomes 6p25 and 4q25, respectively. A third locus for ARS was mapped to chromosome 13q14 but the gene has not yet been identified. Therefore ARS has to be considered as a morphologically [1] and genetically [6] heterogeneous disorder. 

In recent years there has been increasing focus on the clinical as well as a the molecular-genetic aspects of ARS. However, there is an absence of detailed description of the craniofacial and dental manifestations in patients with PITX2 mutation.

## 2. Materials and Methods

In the present multidisciplinary clinical and genetic study of the pedigree four patients with ARS were examined by an ophthalmologist, a dentist, and an orthodontist. 

**Figure 1:**
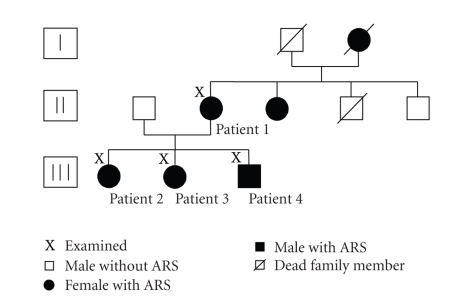
Three-generation pedigree of the reported family.

A three-generation pedigree of the family members was constructed (Figure 1). All four patients were screened for mutations in PITX2 and FOXC1 by direct DNA sequencing [7]. 

The craniofacial and dental examination of the patients involved lateral cephalometric radiographs and orthopantomograms. Analysis of plaster casts was made. The cephalograms were taken with the patients in a cephalostat with ear rods and a light source for adjustment of the head posture. The film-focus distance was 150 cm and the distance from the midsagittal plane to the film was 10 cm, resulting in an enlargement factor of 15%. Angular and linear measurements were made from each cephalogram. The position of the maxilla and mandible Was analyzed by the respective SNA and SNB angles. The skeletal configuration was defined by the Wits appraisal (the AB/Occlusal Plane angle). A metric analysis was done for the length of the maxilla and the mandible. The calibrated cephalometric results were compared to standards of a mean population group by Rakosi [8].

## 3. Results

All four family members showed a sella turcica bridge combined with a prominent posterior clinoid process followed by a steep clivus and an elongated sella turcica, described by Meyer-Marcotty et al. [9].

### 3.1. DNA Analysis

Sequence analysis revealed a variant in the *PITX2* gene. Our four patients were found to have a heterozygous C to T transition at nucleotide position 774. This missense change P64L has already been described in ARS [10].

### 3.2. Patient Reports

#### 3.2.1. Patient 1

**Patient's History**

The patient is 51 years old and a female. ARS was diagnosed at the age of 28 due to impaired vision by the patient herself.

**Ophthalmologic and General Examination**

**Figure 2:**
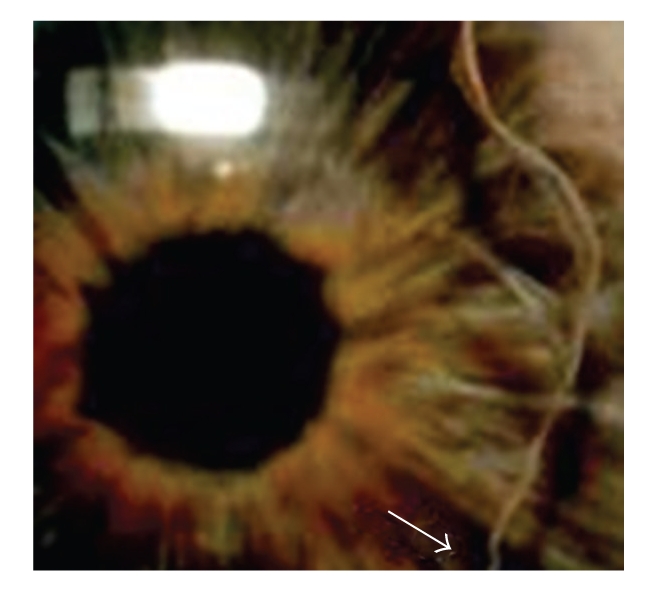
Embryotoxon posterius (↑) and hypoplasia of the iris in patient 1.

The patient presented with embryotoxon posterius, iridocorneal adhesions, and hypoplasia of the iris with the sphincter muscle visible in both eyes (Figure 2). She suffers from glaucoma with a visual field loss of Stage II according to Aulhorn stage classification [11]. The highest recorded intraocular pressure (IOPmax) was 36 mmHg in the right eye and 20 mmHg in the left eye. The patient has a protuberant umbilicus.

**Dental and Craniofacial Findings**

The patient's dental history revealed agenesis unspecified teeth as well as a conspicuous conical shape of the upper permanent incisors. No orthodontic treatment had been undertaken in the past. After additional loss of permanent teeth removable partial dentures were incorporated in the upper and lower jaw.

Extraoral examination of the patient showed a pronounced retrusive lip profile.

Cephalometric analysis indicated skeletal Class III malformation due to severe maxillary retrognathia. Metric analysis of the jaws showed a highly shortened maxilla (micrognathia). The measurements of the mandible were in normative range (Table 1).

#### 3.2.2. Patient 2

**Patient's History**

This 24-year-old female patient was diagnosed with ARS positive during a routine check in early childhood due to the knowledge of the familial predisposition of ARS and glaucoma.

**Ophthalmologic and General Examination**

Embryotoxon posterius, iridocorneal adhesions, Iris hypoplasia, and corectopia are evident in both eyes. There is no record of elevated IOP. The patient has a protuberant umbilicus.

**Dental and Craniofacial Findings**

**Figure 3:**
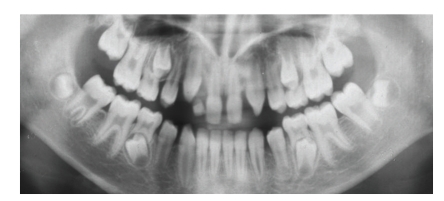
OTP (11 years 3 months) showing agenesis of the upper lateral incisors, taurodontism of the molars, and microdontia with crown hypoplasia of the upper central incisors and congenital missing 18 and 28 in patient 2.

Orthopantomograms taken at the age of 11 years 3 months and 16 years 6 months were available for examination. Agenesis of the upper lateral incisors, taurodontism, microdontia as well a crown hypoplasia of the upper central incisors was present (Figure 3). According to her sister a significant tendency for root resorption was evident after orthodontic treatment.

The cephalometric analysis showed parameters in normative range (Table 1).

#### 3.2.3. Patient 3

**Patient's History**

This 26-year-old female patient suffers from ARS-related ocular hypertension. Her condition was diagnosed at the age of 17 when she went for an examination to an ophthalmologist because of diminished vision which she noticed herself.

**Ophthalmologic and General Examination**

**Figure 4:**
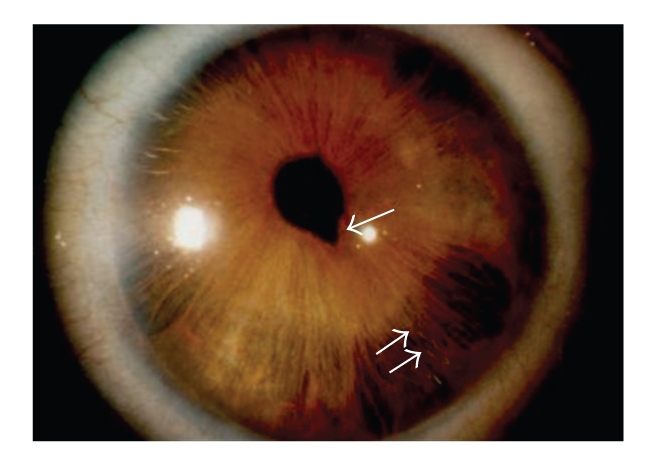
Corectopia (↑) and hypoplasia (↑↑) of the iris in patient 3.

An ophthalmologic examination identified embryotoxon posterius, iridocorneal adhesions, corectopia, and hypoplasia of the iris visible in both eyes (Figure 4). IOPmax was 35 mmHg in both eyes and medical regulation proved to be sufficient. There is no loss of visual field evident up to date. The patient has a protuberant umbilicus.

**Dental and Craniofacial Findings**

**Figure 5:**
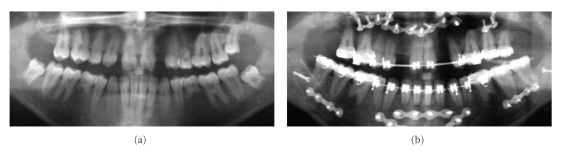
(a) OTP (15 years) showing agenesis of 18 and 15,12, 22, 23, 25, 35, 45; hypoplasia of the crowns of the upper central incisors in patient 3. (b) Severe root resorptions of the lower incisors and the lower first molars in patient 3 after correction of the skeletal Class III relationship by means of combined orthodontic-orthognathic therapy.

The orthopantomogram at the age of 15 revealed agenesis of multiple permanent teeth in association with hypoplastic upper central incisors. Besides agenesis of the second premolars of the upper and lower jaw, the upper lateral incisors were not present. Moreover, a very rare agenesis of the upper left canine was evident (Figure 5(a)).

After correction of the skeletal Class III relationship by means of combined orthodontic-orthognathic therapy the orthopantomogram showed a tendency for root resorption on almost any tooth with a significant reduction of half of the root length of the lower incisors and the lower first molars (Figure 5(b)).

**Figure 6:**
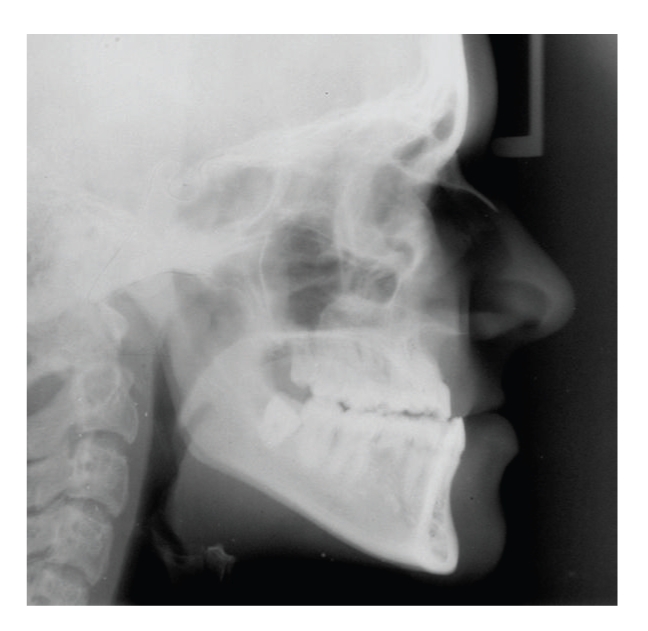
Lateral cephalogram of patient 3 showing a skeletal Class III relationship.

The pretherapeutical cephalometric analysis at 15 years of age revealed a normal sagittal position of the mandible (Table 1). In contrast, the maxilla showed a significant retrognathia resulting in a severe skeletal Class III relationship (Figure 6). As addition, the length of the upper jaw was shortened.

#### 3.2.4. Patient 4

**Patient's History**

This patient is a male and 18 years old. As with his sister, ARS was diagnosed during a routine check in early childhood.

**Ophthalmologic and General Examination**

**Figure 7:**
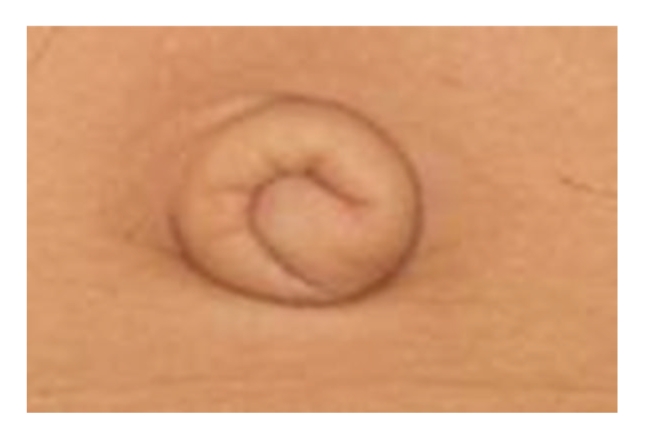
Protuberant umbilicus in patient 4.

Clinical examination identified embryotoxon posterius, iridocorneal adhesion,s and Iris hypoplasia in both eyes. No elevated IOP is present at the moment. The patient has a protuberant umbilicus (Figure 7).

**Dental and Craniofacial Findings**

**Figure 8:**
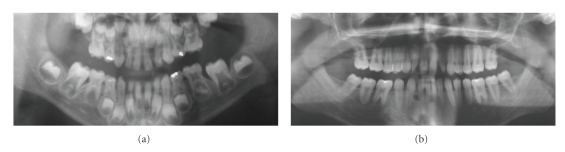
(a) OPT (6 years and 4 months) showing agenesis of upper molars in patient 4. (b) OPT (18 years) showing taurodontism of upper and lower molars and paraplasia on the distal aspect of the right upper third molar in patient 4.

Orthopantomograms taken at the age of 6 years 4 months and 18 years were available for examination. Besides agenesis of the lower right second premolar, the orthopantomogram showed a very rare instance of two missing upper molars (Figure 8(a)). The comparison of the developmental stages between the upper and lower teeth indicated agenesis of the upper first molars. Moreover, taurodontism of the upper and lower molars with variable degree was evident, as well as a paraplasia on the distal aspect of the upper third molar root (Figure 8(b)).

The patient showed conical shaped lateral and central incisors and hypoplastic molar crowns.

The cephalometric measurement revealed a severe skeletal Class III, as seen by a decreased SNA angle combined with a normative SNB. Metric analysis of the jaws showed a shortened maxilla, and an almost normative long mandible (Table 1).

## 4. Review of the Literature

In twelve published case reports [2, 12–22] descriptions of the dental and craniofacial phenotype of 17 ARS patients who were examined by an ophthalmologist and a dentist are given. 

When the four cases examined in the present study are included, 21 patients have been evaluated up to now. 

All the patients presented with hypodontia. These findings were documented in either the primary or permanent dentition depending on the age of the patient. 

**Figure 9:**
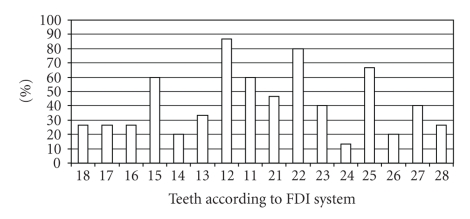
Frequency of agenesis of permanent upper teeth in 15 patients with ARS and hypodontia.

**Figure 10:**
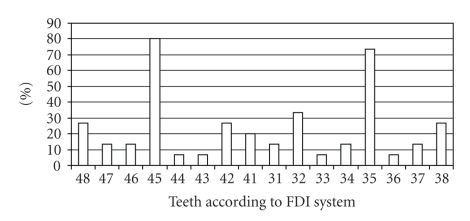
Frequency of agenesis of permanent lower teeth in 15 patients with ARS and hypodontia.

In 15 out of the 21 cases a detailed description of agenesis of permanent teeth was available. In the remaining 6 cases only the total number of missing teeth was reported, ranging from 3 to as many as 20. The teeth most frequently missing were the upper lateral incisors as well as the upper and lower second premolars. Other teeth whose absence was reported were central incisors, canines, first premolars, and first and second molars. Teeth of the upper jaw tended to be missing more frequently (Figures 9 and 10). 

Data on congenital absence of primary teeth were available in 11 out of 21 cases. The teeth most often absent were the upper and lower deciduous incisors, missing in 40% and 18% of the patients, respectively. 

Microdontia has been observed in 17 patients. In 6 patients only maxillary teeth were affected, in 11 patients teeth in both jaws were hypoplastic. Three patients displayed generalized microdontia of all teeth. The teeth series most often affected was the upper and lower incisors, followed by premolars, canines, and molars. Other dental anomalies reported included taurodontism, enamel hypoplasia, short roots, paraplasia, and delayed eruption. 

Maxillary hypoplasia was present in 19 out of 21 patients (90.5%), resulting in a Class III facial profile combined with a flat midface, concave facial profile, receding upper lip, or prominent lower lip.

## 5. Discussion

Axenfeld-Rieger Syndrome is a very rare condition. Thus, the number of patients studied is limited. In addition, this disease shows morphologic variability in terms of frequency and expression of associated anomalies [1, 5, 6]. Therefore, a detailed presentation of dental and craniofacial anomalies in patients with ARS is necessary. In our previous study of 26 patients with ARS and glaucoma or ocular hypertension, dentofacial anomalies were evident in 27% of the patients [3]. This is consistent with the studies by Shields et al. [1] and Ozeki et al. [5] on 24 and 21 patients with ARS, respectively. 

Our patients show a missense mutation in the PITX2 gene. PITX2 is a transcription factor controlling the expression of other genes during development. This factor is essential for correct differentiation and migration of cells developing tissues and organs. Expression of PITX2 in mice has been found in periocular mesenchyme, maxillary, mandibular and dental epithelia, umbilicus, Rathke's pouch, and vitelline vessels [23]. 

PITX2 has been identified as an activator of the Dlx2 gene that is also expressed in maxillary and mandibular and dental epithelia. Dlx2 is part of the “odontogenic homeobox code” essential for tooth and craniofacial development [24]. Therefore dental anomalies in ARS patients can be explained by PITX2 mutation [25]. Depending on the transactivation ability of a PITX2 mutant different phenotypes from mild Iris hypoplasia to the whole spectrum of ARS including severe dental and craniofacial anomalies have been observed [26]. 

In normal Caucasian population hypodontia occurs in males with an incidence of 4.6%, in females of 6.3%, respectively [26]. The teeth most often absent are the lower second premolars followed by the upper lateral incisors and the upper second premolars with a frequency of 1.5%–3.1% [27]. By contrast, all of our ARS patients presented with hypodontia. Moreover, two of the siblings exhibited very rare types of tooth agenesis, such as of an upper canine in patient 3 and of upper molars in patient 4, respectively. 

Microdontia also seems to be a characteristic finding in ARS [2]. In all of our patients a conical shape of the upper central incisors was reported. Additionally, in patient 4 microdontic lower molars were found which is a very rare condition in general population with an incidence of 0.1%–0.4% [27]. 

Taurodontism occurs in approximately 0.3% of the white European population [27]. In patient 4 all molars were affected. This finding supports the hypothesis of previous reports that taurodontism is one of the characteristic dental anomalies in ARS [2, 5]. 

The observed root resorption in two of the siblings may indicate a higher risk of root resorption in ARS patients during orthodontic treatment. 

The four presented cases underwent cephalometric analysis in order to evaluate the craniofacial morphology. 

Three of the patients exhibited a severe skeletal Class III malocclusion caused by maxillary retrognathia associated with a shortened maxilla. In contrast, mandibular position was in an almost normal range. 

The extent of skeletal Class III in our ARS patients was similar to the severity of hypodontia and other dental anomalies. Patient 3 with an agenesis of seven permanent teeth exhibited the most distinctive dysgnathia, whereas patient 2 with agenesis of the upper lateral incisors exhibited almost orthognathic jaw relationships. Drum et al. [14] concluded that an alveolar hypoplasia resulting from missing teeth contributes to a maxillary deficiency. In the present study it seems that the number of missing teeth may play a certain role in the extent of maxillary hypoplasia. However, the reduced maxillary length deficiency seen in our ARS patients is not exclusively limited to the alveolar region but also is evident in the maxillary base. Therefore, maxillary hypoplasia may not completely be explained by the number of congenitally absent teeth. It can be assumed that additional craniofacial factors may influence maxillary growth in ARS patients. Childers and Wright [2] also hypothesied that midface hypoplasia associated with ARS is a combination of skeletal and dentoalveolar factors. 

Early diagnosis of ARS and control of IOP is essential to prevent visual impairment. Ophthalmologists, paediatricians, dentists, and orthodontists have to be aware that ARS is an inherited autosomal dominant anomaly and therefore all family members have to be screened as early as possible. Despite this fact, ARS frequently is diagnosed late. Even hypoplasia of the iris, visible without special instruments, does not reduce the mean age at diagnosis [3]. In addition, two of the patients included in this study were diagnosed only after symptoms of elevated IOP were noticed by the patients themselves.

## 6. Conclusion

Axenfeld-Rieger syndrome is a very rare disorder with genetic and morphologic variability. The concomitant occurrence of rare dental and craniofacial anomalies in these patients, such as hypoplasia of the central incisors, rare types of tooth agenesis such as an upper canine or upper molars associated with maxillary retrognathia, and a skeletal Class III may be indications of ARS. Early detection of ARS and measuring potentially elevated intraocular pressure helps physicians to prevent visual field loss.

The data used in this report are based on the dissertation “Phänotyp und Genotyp des Axenfeld- Rieger- Syndroms” by P. Dressler, Department of Ophthalmology, University of Wuerzburg, Germany (URL: http://www.opus-bayern.de/uni-wuerzburg/volltexte/2006/1850/).

## Figures and Tables

**Table 1:** Cephalometric parameters of the four reported patients. Comparison with normative values according to Rakosi [8].

	Patient 1 (51y)			Patient 2 (22y)			Patient 3 (15y)			Patient 4 (18y)		
Variables	Normative	Measured	Difference	Normative	Measured	Difference	Normative	Analysis	Difference	Normative	Measured	Difference
	Value*	Value		Value*	Value		Value*			Value*	Value	
SNA (°)	81,0 ± 2	71,4	−9,6	81,0 ± 2	81,5	+0,5	81,0 ± 2	78,3	−2,7	81,0 ± 2	77,0	−4,0
SNB (°)	78,0 ± 3	82,6	+4,6	78,0 ± 3	80,9	+2,9	78,0 ± 3	82,2	+4,2	78,0 ± 3	79,8	+1,8
Wits (mm)				0,0 ± 2	−0,1	−0,1	0,0 ± 2	−7,3	−7,3	0,0 ± 2	−2,9	−2,9
Maxillary Length (mm)	53,1	40,7	−12,4	54,9	50,6	−4,3	41,0	36,0	−5,6	34,7	28,9	−5,8
Mandibular Length (mm)	79,7	75,9	−3,8	82,4	87,0	+4,6	62,4	60,4	−2,0	52	50,5	−1,5
